# A Comprehensive Review on Shiga Toxin Subtypes and Their Niche-Related Distribution Characteristics in Shiga-Toxin-Producing *E. coli* and Other Bacterial Hosts

**DOI:** 10.3390/microorganisms12040687

**Published:** 2024-03-28

**Authors:** Xuan Wang, Daniel Yu, Linda Chui, Tiantian Zhou, Yu Feng, Yuhao Cao, Shuai Zhi

**Affiliations:** 1 School of Public Health, Ningbo University, Ningbo 315000, China; wangx317322119@163.com (X.W.); zlabztt@163.com (T.Z.); zlabfy@163.com (Y.F.); 2 School of Public Health, Univeristy of Alberta, Edmonton, AB T6G 2R3, Canada; dyu4@ualberta.ca; 3 Alberta Precision Laboratories-ProvLab, Edmonton, AB T6G 2J2, Canada; linda.chui@albertaprecisionlabs.ca; 4 Department of Laboratory Medicine and Pathology, University of Alberta, Edmonton, AB T6G 2B7, Canada; 5 School of Basic Medical Sciences, Ningbo University, Ningbo 315000, China; caoyuhao@nbu.edu.cn

**Keywords:** Shiga toxin, subtype, host distribution characteristic, prevalence

## Abstract

Shiga toxin (Stx), the main virulence factor of Shiga-toxin-producing *E. coli* (STEC), was first discovered in *Shigella dysenteriae* strains. While several other bacterial species have since been reported to produce Stx, STEC poses the most significant risk to human health due to its widespread prevalence across various animal hosts that have close contact with human populations. Based on its biochemical and molecular characteristics, Shiga toxin can be grouped into two types, Stx1 and Stx2, among which a variety of variants and subtypes have been identified in various bacteria and host species. Interestingly, the different Stx subtypes appear to vary in their host distribution characteristics and in the severity of diseases that they are associated with. As such, this review provides a comprehensive overview on the bacterial species that have been recorded to possess *stx* genes to date, with a specific focus on the various Stx subtype variants discovered in STEC, their prevalence in certain host species, and their disease-related characteristics. This review provides a better understanding of the Stx subtypes and highlights the need for rapid and accurate approaches to toxin subtyping for the proper evaluation of the health risks associated with Shiga-toxin-related bacterial food contamination and human infections.

## 1. Introduction

Shiga toxin (Stx) was first discovered in 1897 in *Shigella dysenteriae* by the Japanese microbiologist Kiyoshi Shiga [1]. Although this toxin initially appeared to be exclusive to *S. dysenteriae* serotype-1 strains, in 1977, Konowalchuk et al. identified a toxin produced by several *E. coli* strains exhibiting toxic effects in Vero cells that was later characterized as a Shiga-like toxin due to its functional, antigenic, and structural similarities to the Stx produced by *S. dysenteriae* [2,3]. Additional commonalities between the identified verotoxin/Shiga-like toxin and Stx were observed at the sequence level, as the translated amino acid sequences for each toxin’s subunits were found to be very similar and, in some cases, even 100% identical [4]. As such, due to the similarity shared between these toxins, the term ”Shiga toxin” was proposed as the prototypical name representing all members of this toxin family, though ”verotoxin” remains in use, albeit with declining popularity [5,6].

Shiga toxin is encoded in the genome of bacteriophages, which is usually integrated as a prophage in the bacterial chromosome [7]. Upon induction, the phage enters the lytic cycle, during which the Shiga toxin gene is expressed [7]. Structurally, Stx is an AB_5_-type toxin composed of one 32 kDa A subunit covalently bonded to a pentamer consisting of five identical 7.7 kDa B subunits [8]. The B subunits target the glycolipid globotriaosylceramide (Gb3) or globotetraosylceramide (Gb4) receptors of host cells [9]. Once bound, Stx can enter host cells, where the N-glycosidase activity of the A subunit leads to the removal of an adenine from the 28S rRNA [10], resulting in the inhibition of protein synthesis in the host cell [8]. Stx can also lead to the activation of ribotoxic stress and endoplasmic reticulum stress responses, which can induce cell death via apoptosis, leading to the increased expression of pro-inflammatory cytokines and the release of reactive oxygen metabolites that lead to endothelial cell injury [11].

While a variety of bacterial species carry *stx* genes, Shiga-toxin-producing *E. coli* (STEC) represents the most common bacterial population that is capable of causing human disease. With ruminant animals as the natural reservoirs for STEC, this pathogen can be transmitted to humans via fecal–oral routes through the ingestion of contaminated food or water [12]. Upon infection, the patient may experience mild to severe diarrhea, hemorrhagic enteritis, or hemolytic uremic syndrome (HUS), resulting in kidney failure, or even death [8,13,14]. STEC strains are estimated to cause over 2,801,000 acute infections worldwide, of which 3890 progress to HUS and 230 result in death [15]. Indeed, STEC infection was consistently ranked as the third most common foodborne pathogen in the U.S. from 2017 to 2022 (https://wwwn.cdc.gov/foodnetfast/, accessed on 2 August 2023), and it continues to impose a significant burden on human health.

At present, a variety of Stx variants have been identified in various bacteria and host species, with each being classified as a different subtype according to their biochemical and molecular characteristics. Interestingly, select Stx subtypes appear to exhibit host-related distribution patterns; thus, a better understanding of the health risks and host-distribution patterns associated with each subtype would greatly inform clinical treatment, risk evaluation, and policy development efforts, especially in the midst of an outbreak caused by an Stx-producing pathogen. Herein, we provide a comprehensive overview on the range of Stx subtypes, including those that were most recently discovered, and explore the bacterial and animal host-related distribution patterns associated with each subtype.

## 2. Classification of Stx1 and Stx2 Subtypes

Shiga toxin can be grouped into two types, Stx1 and Stx2, according to their biological activity and sequence identity [6,8]. Although both toxin types share the same cell receptor and are structurally similar, Stx2 is substantially more toxic than Stx1 as it exhibits higher affinity for host cell ribosomes and displays greater catalytic activity when assessed in cytotoxicity tests using Vero and HeLa cells [16,17,18]. Furthermore, while related, the amino acid sequences of Stx1 and Stx2 are only approximately 56% similar, with each group containing several additional subtypes [11]. In 2012, Scheutz et al. performed a comprehesive sequence-based analysis of Shiga toxin genes and standardized the nonmenclature of the three Stx1 and seven Stx2 subtypes based on their common sequence characteristics [6]. Since then, a couple of new Stx subtypes have been discovered, and to date, Stx1 encompasses four subtypes (*stx*_1a_, *stx*_1c_, *stx*_1d_, and *stx*_1e_), while fifteen subtypes (*stx*_2a_ to *stx*_2o_) are associated with Stx2. Building on this, we performed a comprehensive review on all Stx subtypes that have been discovered to date through scientific literature searches using the keywords “Shiga toxin”, “Verotoxin”, “Shiga-like toxin”, and “subtype”. We also listed the scientific literature in which each Stx subtype was first reported, based on the subtyping protocol from Scheutz et al. [6], and summarized each subtype’s amino acid and nucleotide sequence similarity to the other subtypes when available.

### 2.1. Discovery History of Stx Subtypes

#### 2.1.1. Stx1 Subtypes

The Stx1 subtypes appear to exhibit a high degree of amino acid sequence similarity, which can range from 95% to 98.3% across the different Stx1 subtype groups [6]. Despite this homogeneity, Stx1 can still be classified into four distinct sub-groups, including *stx*_1a_, *stx*_1c_, *stx*_1d_, and *stx*_1e_ (Table 1), on the basis of sequence similarity and differences in each subtype’s biochemical characteristics [6]. As the prototypical-type toxin in the Stx family, the Stx identified in *S. dysenteriae* type-1 strains belong to *stx*_1a_ subtype [6]. Since its discovery, various other Stx toxins belonging to the *stx*_1a_ subtype have been identified in other bacterial species, with it appearing particularly widespread in STEC strains.

The first Stx1 variant, now designated the *stx*_1c_ subtype, was identified by Zhang et al. in 2002 [19]. Compared to the prototypical subtype *stx*_1a_, this subtype was found to exhibit 97.1% and 96.6% amino acid sequence identity, respectively, in its A and B subunits. Beyond sequence divergence, the authors also demonstrated that Stx1c toxins may be antigenically distinct from Stx1a toxins, as Stx1c toxins were titered out to a significantly lesser degree than Stx1a toxins in a verotoxin-producing *E. coli* reverse passive latex agglutination (VTEC-RPLA) assay [19].

In 2003, Bürk et al. [20] identified another novel Stx1 variant, designated as *stx*_1d_. Compared to the prototypical *stx*_1a_, *stx*_1d_ only exhibited 91% nucleotide identity overall, while sharing 93.7% and 92.1% amino acid identity in their A and B subunits, respectively. Furthermore, the Stx1d toxins appeared to be less potent compared with their prototypical counterpart, as Stx1d toxins exhibited substantially lower levels of cytotoxicity to Vero cells than Stx1a toxins [20].

The final Stx1 subtype, *stx*_1e_, was discovered in 2014 by Probert et al. [21] after Shiga toxin was detected in an *Enterobacter cloacae* strain isolated from an HUS patient from whom the supernatant of a culture of *E. cloacae* strain was found to be cytotoxic to Vero cells. Sequence analysis demonstrated that the *stx* toxin shared only 87% amino acid sequence identity with the closest Stx1 subtype reference sequence, thereby prompting its designation as a new subtype, *stx*_1e_ [21].

#### 2.1.2. Stx2 Subtypes

Stx2 toxins only exhibit roughly 50–60% amino acid identity compared to their Stx1 counterparts [31]. Furthermore, whereas Stx1 toxins exhibit a relatively high degree of sequence homogeneity across subtypes, Stx2 toxins are considerably more heterogeneous. Currently, fifteen Stx2 subtypes (*stx*_2a_ to *stx*_2o_) have been reported (Table 1), based on differences in their nucleotide/amino acid sequence identity [35].

The origins of Stx2 toxins date back to 1982, when Riley et al. first recovered *E. coli* O157:H7 isolates from the stool samples of patients experiencing bloody diarrhea during an outbreak [36]. While the mechanism of action was unknown, the authors suggested that the recovered *E. coli* isolates were causing the episodes of bloody diarrhea through the production of a toxin that had not yet been identified. In the same year, Johnson et al. reported for the first time that *E. coli* O157:H7 strains could be producers of the Vero cytotoxin [37]. It was not until a few years later that Strockbine et al. [38] identified two distinct Shiga-like toxins from *E. coli* O157:H7 strains in 1986 and named them Shiga-like toxin I and Shiga-like toxin II, respectively. Specifically, the toxin that was eventually named Shiga-like toxin II now serves as the prototypical Stx2 toxin, and it has been confirmed to belong to the *stx*_2a_ subtype based on sequence homology [22].

The first nucleotide sequence of an *stx*_2b_ toxin was reported by Paton et al. in 1992 [23], which was derived from an Stx produced by an *E. coli* OX3:H21 strain recovered from an infant that passed from Sudden Infant Death Syndrome. Nucleotide sequence analyses revealed that the A subunit of this toxin exhibited 95.9% sequence identity to a previously-identified *stx*_2d_ subtype, whereas the B subunit only exhibited 88.6% similarity to its Stx2d counterpart [23].

In 1991, the first *stx*_2c_ sequence was reported by Schmitt et al. [24], which was derived from a toxin recovered from an *E. coli* O157 strain. Comparative nucleotide sequence analyses revealed that the A and B subunit sequences of the *stx*_2c_ subtype shared anywhere between 94.6%~98.5% and 79.0~98.6% identity with the toxins beloninging to the *stx*_2a_, *stx*_2d_, and *stx*_2e_ subtypes.

The first Stx toxin belonging to the *stx*_2d_ subtype appears to have been discovered in 1990 by Ito et al. [25], which was based on the characterization of a toxin originally named VTX2ha that was produced by an *E. coli* O91:H21 strain recovered from an HUS patient. Nucleotide sequence homology analyses demonstrated that this *stx*_2d_ subtype shared 98.6% and 95.5% nucleotide identity with the A and B subunits of an *stx*_2a_ subtype, respectively. Furthermore, when compared to other Stx2 variants, *stx*_2d_ was found to share anywhere between 94.5%~99% and 81.5%~96% identity at their A and B subunits, respectively [39].

In 1987, Marques et al. isolated the first *stx*_2e_ subtype from swine with edema disease. Unlike other Stx subtypes, *stx*_2e_-encoded toxins were found to be cytotoxic to Vero cells, but not to HeLa cells [40,41]. Furthermore, comparison of the Stx2 toxins SLT-II and SLT-IIv, which belong to the *stx*_2a_ and *stx*_2e_ subtypes, respectively, revealed a high degree of nucleotide sequence identity (94%) in the A subunit but significantly greater sequence divergence in the B subunit (79%) [26], thereby justifying its designation as a distinct Stx2 subtype.

In 2000, Schmidt et al. [27] isolated a STEC O128:H2 strain from pigeon feces carrying an Stx variant that was eventually determined to belong to the *stx*_2f_ subtype. This Stx variant reacted weakly to commercial Stx immunoassay tests, and was found to only share 63.4% and 57.4% sequence homology at the A and B subunits, respectively, when compared to an *stx*_2a_ counterpart [27]. Interestingly, the toxin variant characterized by Schmidt et al. was found to instead share high sequence similarity with a different Stx toxin, designated as sltIIva, that was derived from a STEC strain recovered from a human infant with diarrhea by Gannon et al. in 1990 [42]. These two toxins shared 99.8% and 100% sequence identity at their A and B subunits, respectively, indicating that the Stx variant identified by Gannon et al. [42] could be the first published Stx sequence belonging to the *stx*_2f_ subtype.

The *stx*_2g_ subtype was first identified by Leung et al. [28] in 2003, through the characterization of an Stx toxin produced by an *E. coli* O2:H25 strain isolated from the feces of healthy cattle. While this strain displayed a similar level of cytotoxicity to Vero and Hela cells when compared to an ATCC 43889 strain producing both Stx2a and Stx2c toxins, the toxin it produced appeared to be quite divergent from other known Stx subtypes at the time. Reflecting this, compared to several previously reported Stx variants representative of the subtypes from *stx*_2a_ to *stx*_2f_, the nucleotide identity shared at the A and B subunits ranged from 63.0~94.9% and 76.7~90.7%, respectively [28].

In 2018, Bai et al. [29] characterized an Stx2 toxin belonging to the *stx*_2h_ subtype in a STEC O102:H18 strain isolated from marmots. While this toxin was found to exhibit similar cytotoxicity in Vero cells compared to the Stx produced by an *E. coli* O157:H7 strain, other metrics confirmed its divergence as a novel Stx2 subtype. In terms of sequence homology, this Stx2h toxin was found to exhibit anywhere from 69.7~92.9% and 67.2–91.3% identity in its A and B subunit, respectively, compared to representative Stx2 toxins belonging to the other subtypes from *stx*_2a_ to *stx*_2g_ [29]. Furthermore, the closest Stx2 subtype to the Stx2h toxin was only found to share 91.9% and 92.9% nucleotide and amino acid identity, respectively. The divergent nature of the *stx*_2h_ subtype was further indicated through phylogenetic analyses, as strains producing Stx2h toxins formed a distinct cluster amongst all other strains analyzed, further prompting the designation of this toxin as a novel Stx2 variant [29].

The *stx*_2i_ subtype was identified in 2016, when Lacher et al. [30] used an *Escherichia coli* identification microarray to characterize a set of STEC strains isolated from food sources. Interestingly, several strains were identified as belonging to *stx*2 of an unknown subtype, including select *E. coli* ONT:H25 strains isolated from shrimp that were found to carry the newly-named *stx*_2i_ subtype gene [30].

In 2022, Gill et al. [31] identified a new Stx2 subtype, designated as *stx*_2j_. The *stx*_2j_ subtype was first identified in two STEC strains, including one *E. coli* O158:H23 strain isolated from lettuce and one human-derived *E. coli* O33:H14 strain. Sequence comparisons revealed that the Stx2j toxins produced by these two strains were identical at the sequence level, and both exhibited cytotoxicity in Vero cells. Furthermore, compared to the other Stx2 subtypes, this novel *stx*_2j_ subtype was found to be closest to *stx*_2h_, sharing up to 91.9% nucleotide sequence identity [31]. The unique phylogenetic clustering of the *stx*_2j_-positive *E. coli* strains, however, including the two described above and an additional STEC strain isolated from human fecal samples, supports the designation of these toxins as a novel Stx2 subtype [31].

The *stx*_2k_ subtype was first identified by Yang et al. [32] in 2020 through a survey of various *E. coli* strains recovered from a variety of sources, including diarrheal patients, various animals, and raw meat samples. In particular, nine Stx2-producing strains were found to exhibit variable levels of cytotoxicity in Vero cells, with one strain exhibiting low cytotoxicity that was comparable to a negative control. Furthermore, when these strains were compared through an Stx production assay, only five produced a strong band, whereas the remaining four only produced weak or no bands, suggesting that each strain varied in the amount of Stx2k produced. Futhermore, they belonged to different serogroups, such as O48, O159, O100, O22, and O93. At the sequence level, these Stx2k toxins were found to share anywhere between 46.3~98.3% and 69.9~100% nucleotide sequence identity compared to reference toxins belonging to the other Stx2 subtypes from *stx*_2a_ to *stx*_2i_ [32].

In 2019, Koutsoumanis et al. [33] re-designated a specific Stx2e toxin, Stx2e-O8-FHI-1106-10, as belonging to a novel subtype, *stx*_2l_. This Stx2e-O8-FHI-1106-10 toxin was originally discovered by Lindstedt et al. in Norway in 2007 and has since been identified in some human diarrhea cases, being recovered from *E. coli* strains belonging to various serotypes, including O8:H9, O8:H19 and O8:H30. Interestingly, this *stx*_2l_ subtype was also identified in *E. coli* O8:H30 strains collected from diarrheal patients in Denmark, suggesting that toxins belonging to this novel Stx2 subtype could be of growing clinical importance.

The *stx*_2m_ subtype was discovered by Bai et al. [34] in 2021 in three *E. coli* strains that were isolated from diarrheal patients and asymptomatic carriers in Sweden and Denmark. Despite the differences in symptomatic presentation, all three strains, including two O148:H39 strains from Sweden and one O96:H19 strain from Denmark, were found to be cytotoxic to Vero cells. At the sequence level, these Stx2m toxins were found to share anywhere between 63.4~92.6% nucleotide sequence identity and 72.4~93.8% amino acid identity with the twelve other Stx2 subtypes from *stx*_2a_ to *stx*_2l_ [34].

In 2022, Gill et al. [19] first discovered the *stx*_2o_ subtype in a human-derived *E. coli* O85:H1 strain. Detailed characterization of this new subtype was further studied by Lindsey et al. [35] in 2023, during which they found that the *stx*_2o_-positive strain was cytotoxic to Vero cells. In addition, at the sequence level, the *stx*_2o_ subtype was found to share 70.4–94.1% nucleotide sequence identity and 81.8–96.9% amino acid sequence identity with other Stx2 subtypes.

The final Stx2 subtype, *stx*_2n_, was discovered by Lindsey et al. [35] in 2023 in two clinical *E. coli* strains after cytotoxicity tests showed that these *stx*_2n_-positive strains were toxic to Vero cells. At the sequence level, these *stx*_2n_ subtypes were found to share anywhere between 72.2~94.6% nucleotide sequence identity and 83.9~95% amino acid identity with the other Stx2 subtypes.

### 2.2. Prevalence and Disease Severity of Different Stx Subtypes in Humans

Several studies have found that *stx*1 and *stx*2 are equally frequent among STEC-related human infections [43,44]. For instance, in a study assessing 606 clinical STEC strains, up to 42.6% were identified as *stx*1-possessing strains and 36.8% as positive for *stx*2, while 20.6% carried both *stx*1 and *stx*2 [43]. Likewise, Nüesch-Inderbinen et al. [44] found that out of 120 STEC isolates recovered from clinical samples, 36.7% or 44 were *stx*1-positive, 40.0% or 48 were *stx*2-positive, and the remaining 23.3% or 28 had both *stx*1 and *stx*2. Despite the similar degrees of prevalence between *stx*1 and *stx*2, however, *stx*2 is usually more commonly linked with severe disease manifestations (i.e., HUS). For instance, one study estimated that 59.1% of patients infected with *stx*1-positive STEC experienced diarrhea, with none presenting with HUS; in contrast, the authors observed a higher rate of diarrhea in 68.8% of patients infected with *stx*2-positive STEC, of which 12.5% also progressed to HUS [44]. Similarly, in a survey of patients experiencing HUS, De Rauw et al. [43] found that only one case of HUS was caused by a STEC strain producing the Stx1 toxin.

Among the Stx1 subtypes, *stx*_1c_ and *stx*_1d_ are less commonly carried by STEC strains causing human infections than *stx*_1a_ [8]. For instance, an investigation on clinical STEC strains collected across 27 years in a Belgium hospital revealed that 88.4% of STEC infections were caused by exclusively Stx1-producing strains that produced toxins belonging to the *stx*_1a_ subtype [43]. Furthermore, Chattaway et al. [45] determined that 72% of infections caused by Stx1-producing STEC in England involved strains producing toxins that belonged to the *stx*_1a_ subtype. Similar findings were also obtained in a study conducted by Moeinirad et al. [46], in which the authors found that 75% of Stx1-exclusive STEC strains recovered from diarrheal cases in children in Iran produced *stx*_1a_ subtype toxins, whereas the rest produced toxins belonging to the *stx*_1c_ subtype.

While toxin subtype can have a large impact on pathogenicity, the disease severity characteristics associated with STEC strains can be jointly influenced by a variety of virulence factors rather than by Stx alone. Therefore, it is not surprising that STEC strains carrying the same Stx subtype might exhibit significant differences in the severity of diseases that they cause. Despite this, the Stx1 subtypes appear to exhibit differences in their symptom severity, as strains positive for *stx*_1a_ are more commonly associated with severe clinical outcomes compared with strains belonging to the other Stx1 subtypes [47,48]. For instance, in a survey of the clinical outcomes of patients infected with Stx1-producing STEC, Matussek et al. [48] found that 3 out of 31 patients infected with *stx*_1a_-positive strains developed bloody diarrhea compared with none of the 4 patients that were infected with *stx*_1c_-positive STEC. Similarly, between the years of 2003 and 2005 in Sweden, bloody diarrhea was observed in 7 out of 17 patients that were infected with an *stx*_1a_-positive STEC strain and none in any of the 4 patients infected with *stx*_1c_-positive strains [47]. Indeed, though other studies have only identified a slight increase in disease severity between patients infected with STEC producing *stx*_1a_- and *stx*_1c_-subtype toxins [49], *stx*_1a_-positive strains appear to be more closely associated with severe disease. In contrast, strains producing *stx*_1d_-subtype toxins are rarely found in human infections [29,48], and there is limited data available on the clinical manifestations of *stx*_1d_-positive strains. One study, however, did identify one *stx*_1d_-associated infection among 120 patients in Switzerland, resulting in diarrhea and abdominal pain without any progression to HUS [44].

Though Stx2 toxins are usually implicated in severe disease, not all Stx2 subtypes exhibit the same prevalence and symptom severity in human infections. At present, the *stx*_2a_, *stx*_2b_, *stx*_2c_, and *stx*_2d_ subtypes are the most frequently reported Stx2 subtypes in human disease. For instance, in Switzerland, Ferdous et al. [50] found that the *stx*_2c_-subtype toxins were the most common in STEC-associated human infections, followed by those from the *stx*_2a_, *stx*_2b_, and *stx*_2d_ subtypes. In contrast, in two Dutch regions, the most common Stx2 subtypes identified amongst STEC strains were *stx*_2a_ and *stx*_2b_, followed by *stx*_2d_, *stx*_2c_, and *stx*_2e_ [44]. A larger degree of variability in Stx2 subtype prevalence was identified by Carroll et al. [51], where in England, it appeared that *stx*_2b_ was the most common Stx2 subtype found in clinical STEC strains, and it was found at a substantially higher rate than that of the *stx*_2a_, *stx*_2c_, and *stx*_2d_ subtypes. The findings of these studies thus highlight the differences in the geographical and temporal distribution of Stx2 subtypes produced by STEC strains associated with human infections; however, the differential distribution of Stx2 subtypes could be caused by various factors, including different specimen screening strategies or insufficient numbers of isolates surveryed across different studies.

Regarding disease severity, out of all the Stx2 subtypes, *stx*_2a_, *stx*_2b_, *stx*_2c_, *stx*_2d_, *stx*_2e_, and *stx*_2f_ have been found to be associated with severe disease, particularly HUS [43,52,53,54]. Of these subtypes, *stx*_2a_ appears to carry the highest risk of causing HUS. For instance, in the same survey of HUS patients, De Rauw et al. [43] observed that 78.3% of STEC strains recovered from HUS patients were *stx*_2a_-positive. Overall, *stx*_2a_ appears to be more generally associated with severe disease than the other subtypes, appearing in up to 66.7% of STEC isolates associated with bloody diarrhea and HUS [49,50]. Similarly, through a comprehensive survey of 29,945 human STEC infections in the European Union from 2012–2017, Panel et al. [33] found that *stx*_2a_, *stx*_2b_, *stx*_2c_, and *stx*_2f_ were the top four Stx2 subtypes represented, contributing to more than 90% of the severe clinical cases (i.e., HUS, bloody diarrhea, etc.) requiring hospitalization. In contrast, the *stx*_2d_, *stx*_2e_, and *stx*_2g_ subtypes have been rarely identified in human infections, and they are typically linked with only mild disease manifestations. The other subtypes (i.e., *stx*_2h_, *stx*_2i_, *stx*_2j_, etc.), lack validated approaches for detection; thus, further study is required to evaluate their clinical relevance and associated disease severity.

### 2.3. Stx Subtyping Approaches

Given that certain Stx subtypes are more closely associated with severe clinical outcomes than others, the rapid and accurate subtyping of Stx carried by different bacterial pathogens is imperative for effective disease management and public health interventions, especially during outbreak situations. The current gold standard for Stx subtyping is a PCR panel developed by Scheutz et al. [6]. In this PCR panel, primers for the specific amplification of three Stx1 subtypes (*stx*_1a_, *stx*_1c_, *stx*_1d_) and seven Stx2 subtypes (*stx*_2a_~*stx*_2g_) were developed based on 47 Stx1 and 238 Stx2 subtype sequences. The PCR protocol was then validated extensively against 62 reference strains carrying different Stx subtypes, 160 clinical STEC strains, and 42 EHEC strains associated with HUS. This PCR protocol has greatly simplified the Stx subtyping process, as it forgoes the need for any sequencing or bioassays; as a result, it has become the most widely used Stx subtyping approach to date.

Based on the PCR design by Scheutz et al. [6] and the current nomenclature scheme for the Stx subtypes, real-time PCR protocols have been developed by other groups to expedite the subtyping process and make it less labor-intensive. For instance, in 2019, Zhi et al. [55] developed real-time PCR assays for the three Stx1 subtypes and seven Stx2 subtypes included in the gold-standard PCR panel. All assays were probe-based, aside for those designed to detect *stx*_2c_ and *stx*_2d_ as their sequences were too similar for the development of distinct probes and primers. In the assay, a locked nucleic acid (LNA) probe was used, allowing for the design of shorter probe sequences as the hybridization temperature of the LNA probe could be increased through the modification of its ribose backbone. This short probe design was found to be especially useful as longer probe sequences could not be used due to the high degree of sequence homology shared between some of the Stx subtypes. These assays were evaluated against a panel of 39 STEC strains spanning 10 Stx subtypes, and supplemented with in silico analyses comparing the primer and probe sequences against various Stx subtypes deposited in the NCBI database, with both approaches suggesting that the assays developed were sensitive and specific. In 2023, Harada et al. [56] also designed a set of LNA-probe-based, real-time PCR assays for the same three Stx1 and seven Stx2 subtypes. The assays were validated against a collection of 103 STEC strains and were demonstrated to be sensitive and specific for most of the *stx* subtypes, though some crossreactivity was observed between the *stx*_2a_ and *stx*_2b_ assays [56].

In addition to the PCR and real-time PCR assays developed for Stx subtyping, bioinformatic approaches have also been used to determine Stx subtypes based on whole-genome sequencing (WGS) data [45,57]. For instance, Ashton et al. [57] used two complementary methods, de novo assembly and read mapping, to determine Stx subtypes from sequence data. This method was tested on 444 STEC strains and evaluated against the PCR method developed by Scheutz et al. [6], with the bioinformatic-based approach achieving 99% congruency (i.e., 442 out of 444 strains) with the typing results obtained from the PCR method [57]. Given that WGS is being increasingly adopted by clinical laboratories and used for public health surveillance and outbreak investigations, the ever-increasing availability of ready-to-use genome data makes bioinformatic tools a promising approach for the evaluation of Stx subtypes and their associated health risks. Overall, however, among all the subtyping techniques that are currently available, real-time PCR appears to be best suited for time-sensitive situations, especially in assessing risks to patients infected with Stx-producing pathogens to inform disease management strategies. In contrast, the WGS approach would be useful when real-time PCR methods are not available for certain subtypes (i.e., *stx*_2m_, *stx*_2n_, *stx*_2o_). Furthermore, whole-genome data can also provide additional information (i.e., about other virulence factors) that can be used for risk evaluation efforts.

## 3. Shiga-Toxin-Producing Bacteria

Conventionally, *E. coli* and *S. dysenteriae* type-1 strains are considered to be the major carriers of Shiga toxins; however, these toxins have also been described in several other bacterial genera and species, including *Shigella flexneri*, *Shigella sonnei*, *Citrobacter* spp., *Aeromonas* spp., *Acinetobacter haemolyticus*, *Salmonella* spp., *Vibrio vulnificus*, and *Campylobacter* spp. [58,59,60,61,62,63,64,65,66,67], among others, with each of these representing emerging and growing public health risks.

### 3.1. Shigella spp.

The genus *Shigella* contains four species: *S. dysenteriae*, *S. flexneri*, *S. sonnei*, and *S. boydii*. Of these four species, *S. dysenteriae*, specifically strains belonging to serotype 1, were historically recognized as the only members of the *Shigella* genus to produce Shiga toxin. In recent decades, however, clinical *stx*-carrying strains belonging to the other *Shigella* species have been discovered. The first published case of an *stx*-positive non-*S. dysenteriae* serotype-1 strain was reported by Beutin et al. [58], after the isolation of a *S. sonnei* strain from a diarrheal patient that was found to produce Stx1 toxin. Later, in a surveillance study for the presence of Stx in *S. sonnei*, Lamba et al. [59] identified 56 cases of infection caused by Stx1-positive *S. sonnei* strains, among which 71% of patients presented with bloody diarrhea. In recent years, Shiga toxin has also been frequently reported in *S. flexneri*, with most cases related to travel and/or residency in the Carribean (i.e., Haiti and Dominican Republic), with the identified Stx toxins belonging to the *stx*_1a_ subtype [60,68].

Beyond the discovery of Stx in different *Shigella* species, Stx has also been recorded in strains belonging to *S. dysenteriae* lineages aside from serotype 1. For instance, in addition to identifying Stx1-positive *S. sonnei* strains in clinical samples collected from travellers returning from the Carribean regions, Fogolari et al. [69] were also able to isolate serotype-4 *S. dysenteriae* strains that carried Stx1. Similarly, in another study, Gupta et al. [70] documented three patients infected with Stx1-positive *S. dysenteriae* type-4 strains, two of whom presented with bloody diarrhea, that had recently travelled to Haiti, the Dominican Republic, and Punta Cana. As such, various members of the *Shigella* genus beyond *S. dysenteriae* type-1 strains appear to be producers of Stx1. Indeed, the rising prevalence of Stx1-positive *Shigella* spp. circulating in the Carribean countries may represent an emerging public health risk globally, with cases having been identified in travellers from the U.S. [59,70,71,72,73], Canada [74], and France [60].

### 3.2. Escherichia coli

*Escherichia coli* is part of the normal gut flora in humans; however, some strains can be pathogenic, causing a wide spectrum of intestinal or extra-intestinal diseases. Specifically, intestinal pathogenic *E. coli* include the enteropathogenic *E. coli* (EPEC), enterotoxigenic *E. coli* (ETEC), enteroinvasive *E. coli* (EIEC), enterohemorrhagic *E. coli* (EHEC), and enteroaggregative *E. coli* (EAEC). Of these, EHEC is best recognized for its carriage and production of Stx toxins and its ability to cause hemorrhagic enteritis and hemolytic uremic syndrome [13,14,75]. Although EHEC is the pathotype most closely associated with Stx, these toxins can also be found in *E. coli* strains belonging to different pathotypes. For example, during an investigation of an outbreak of HUS in France, Morabito et al. [76] identified the causative agent to be an EAEC strain that was producing Stx. Similarly, the 2011 *E. coli* O104:H4 outbreak in Germany was linked to a hybrid of EAEC and EHEC strains that produced Stx2 toxins [77,78]. Other Stx-producing hybrid strains have also been reported, including EPEC/EHEC [79,80], ETEC/EHEC [80], ETEC/STEC [81], STEC/UPEC (uropathogenic *E. coli*) strains [82].

As a species, *E. coli* is characterized by a substantial level of strain diversity. Generally, *E. coli* strains can be classified by the O- and H-antigens located at their membrane surface. According to EnteroBase (https://enterobase.warwick.ac.uk, accessed on 24 July 2023), 187 O- and 53 H-antigens have been identified [83]. The first-identified STEC was found to be an *E. coli* O157 strain, which is currently known as one of the leading serogroups of STEC causing severe disease in humans [84]. Outside of O157, however, over 100 additional serogroups have also been found to be associated with human illnesses [49]; of these, O26, O103, O111, O121, O45, and O145 have been recognized as among the most common non-O157 serogroups associated with HUS [85,86].

While the non-O157 STEC strains can cause a myriad of diseases, ranging from mild enteric symptoms to severe cases of HUS or even death, the diseases typically associated with non-O157 STEC infections are generally milder compared with those infections caused by O157 STEC strains [86]. For instance, in an analysis comparing the disease severity of non-O157 and O157 infections, Hedican et al. [87] found that 54% and 8% of non-O157 infections led to bloody diarrhea and hospitalizations, respectively, compared with 78% and 34% of O157 infections. Similarly, Gloud et al. [86] found that non-O157 STEC infections were less likely to progress to bloody diarrhea than O157 STEC infections (55% vs. 85%, respectively), and that of these, fewer non-O157 cases required hospitalization compared with those caused by O157 STEC strains (14% vs. 43%, respectively).

### 3.3. Citrobacter spp.

Like *E. coli*, the *Citrobacter* genus also belongs to the *Enterobacteriaceae* family, consisting of aerobic Gram-negative rod-shaped bacteria with a wide distribution across a variety of external environments (i.e., water, soil, sewage) that also form part of the normal intestinal flora of animals and humans [88]. As such, *Citrobacter* is considered to be an opportunistic pathogen in humans and can cause a range of infections including urinary tract infections, gastroenteritis, and meningitis [88]. The first recorded case of Stx within *Citrobacter* was in 1992, when Schmidt et al. [61] screened 928 human fecal samples and 51 beef samples and identified seven *C. freundii* strains that carried an *stx*_2c_ gene. Later, in 1995, epidemiological investigations of an outbreak at a nursery school in Germany that resulted in nine cases of HUS and one death revealed the causative agent to be a *C. freundii* strain capable of producing Stx2-type toxins [89]. Recently, in 2020, the first case of an Stx1-positive *Citrobacter* strain was recorded after it was linked to two cases of diarrhea in Iran during an outbreak [90].

### 3.4. Enterobacter cloacae

*Enterobacter cloacae* is a Gram-negative facultative anaerobic bacterium that is a normal resident in the animal and human gut [91]. Despite this, *E. cloacae* can cause disease, and it is commonly associated with hospital-acquired infections [92]. In 1996, an Stx2-positive *E. cloacae* strain was isolated from an HUS patient [62]; while PCR, DNA hybridization assays, and Vero cell cytotoxicity assays all confirmed that the strain produced an Stx2-type toxin, the presence of the *stx*2 gene seemed to be unstable as it could not be identified consistently in all subcultures. In 2014, Probert et al. [21] isolated an Stx1-producing strain of *E. cloacae* from a human patient that presented with non-bloody diarrhea and abdominal cramping but no severe symptoms. Unlike the Stx2-positive strain that was originally identified by Paton et al. [62], the presence of the *stx*1 gene was relatively stable as it could still be detected after multiple rounds of subculturing [21].

### 3.5. Aeromonas spp.

The *Aeromonas* genus consists of Gram-negative bacilli that are mainly distributed in aquatic environments. Some strains, however, have been isolated from animals and food sources, while others have also been linked to various diseases in humans, including gastroenteritis and bacteremia [93,94,95]. In 1996, Haque et al. [96] tested the virulence properties of 37 strains of *Aeromonas* spp. Isolated from both diarrheal patients and the environment. Surprisingly, 31 were found to be cytotoxic to Vero cells, including three *A. hydrophila* isolates that were confirmed to be the causative agent of gastroenteritis in diarrheal patients from Thailand and Japan that were later found to be positive for Stx1 by PCR [96]. In a later study conducted in 2000, virulence gene screening of *Aeromonas* spp. Collected from water pipes identified one strain of *A. veronii* biovar sobria that was positive for stx1 [97]. Similarly, using a PCR screening assay to identify *stx* genes in 80 clinical *Aeromonas* strains, Alperi and Figueras [98] identified 19 *A. caviae*, *A. hydrophila*, and *A.beronii* strains that were Stx positive, of which 18 produced only Stx1 toxins, while a lone strain was positive for both Stx1 and Stx2. Importantly, however, the authors found that among the 19 *stx-*positive strains, only 4 consistently generated strong PCR bands, suggesting that the *stx* genes in the remaining 15 strains may have been lost during subculturing.

### 3.6. Other Bacteria

Over the years, *stx* has also been increasingly found in a variety of other bacterial species. For instance, in 2006, Grotiuz [64] reported the first Stx2-positive *Acinetobacter hemolyticus* strain from an infant with bloody diarrhea. Later, in 2014, another Stx-positive *A. hemolyticus* strain was identified as the causative agent of HUS in a 9-month-old infant [65]. Beyond *Acinetobacter*, after screening 278 bacterial isolates that were positive for the *eae* gene, Ooka et al. [66] identified two strains of *E. albertii*, one isolated from a diarrheal patient and the other from a healthy bird, that harbored an *stx*2 gene belonging to the *stx*_2f_ subtype. Two other studies later isolated Stx2-producing *E. albertii* strains causing enteric infections in human patients, including an *stx*_2a_-positive strain in Norway [99] and an *stx*_2f_-positive strain in Brazil [63]. Moreover, the *Campylobacter* genus has also been reported to produce low levels of Shiga toxin; however, this toxin has been shown to be genetically distinct from the Stx toxins produced by *E. coli* [67]. Finally, according to the NCBI pathogen detection database (https://www.ncbi.nlm.nih.gov/pathogens/, accessed on 2 August 2023), other bacterial species have also been found to carry *stx* genes, including a *Salmonella enterica* strain (GenBank Accession: AAAFNG000000000) carrying the complete sequence of an *stx*_2f_ gene that was isolated in the United Kingdom, as well as a *Listeria monocytogenes* strain (GenBank Accession: AAASXN000000000) carrying the complete sequence of an *stx*_1a_ gene that was recovered from animal manure in the United States.

To date, although Stx has been identified in a variety of bacterial species, *E. coli* is still considered the primary contributor to Stx-related infections in humans. Nevertheless, due to the severity of diseases that can be associated with Stx-related infections, non-*E. coli* Stx-positive pathogens still warrant close monitoring as some of these emerging strains could adapt to, and subsequently establish themselves in, the human population, potentially leading to serious outbreaks.

## 4. Host Distribution Patterns of Different Stx Subtypes

Outside of humans, STEC has also been found in a variety of animals hosts, including cattle, sheep, swine, birds, and deer [100]. While STEC is typically pathogenic to human hosts, most animals lack the appropriate Shiga toxin receptors [100], so they do not usually experience disease symptoms upon infection. Despite this, these animals can still act as reservoirs for STEC and can contribute to human infections through the transmission of STEC through fecally-contaminated food and water sources [101,102].

### 4.1. Cattle

Cattle are the most important host reservoirs for STEC. While it was generally believed that the asymptomatic colonization of STEC in cattle was due to the lack of appropriate vascular receptors for Shiga toxins [103], other studies have demonstrated that the expression of Gb3 by crypt epithelial cells of the small and large intestine in cattle occludes the translocation of Shiga toxin which, when combined with the receptor isoform(s) and their organization on these cells, leads to an absence of local or systemic cytotoxicity and overall inflammation in the bovine host [104,105,106]. Typically, cattle hosts can be infected with STEC during the first few months of their life through exposure to contaminated food, water, or other environmental sources or through direct contact with other infected cattle hosts. After infection, STEC will primarily colonize the terminal rectum within the cattle gastrointestinal tract, after which the cattle host may then become a persistent shedder of STEC [107].

Across cattle populations, the average prevalence of STEC can range from 12% to 24% [108,109,110,111]; however, the specific prevalence of STEC can vary widely depending on the specific herd. For instance, long-term studies monitoring cattle herds on four German cattle farms from birth to slaughter revealed that the STEC prevalence ranged from as low as 29% to as high as 82% [112]. Similarly, a survey of 15 cattle farms in Korea demonstrated that the STEC prevalence was highly variable, ranging from 0% to 90% across herds [108].

Given that the prevalence of STEC in a given cattle population can be influenced by many factors, including age, climate, feeding habits (i.e., feedlot or pasture), and sanitation, different herds may be characterized by drastic differences in their STEC carriage [113,114,115]. Reflecting this, calves appear to be more likely to shed STEC during the first six months after birth, after which the shedding of STEC decreases as the calves mature [113,115]. Similarly, other studies have found that the STEC prevalence among cattle is highest at two years of age, and decreases with age [114]. In addition to age, seasonality and temperature are other influencing factors, as there appears to be a significant association between warmer seasons and a higher STEC prevalence [108,111], with prevalences rising in the late spring and peaking around early fall [110,116].

Although cattle are the most common reservoirs for STEC, there appear to be cattle-specific distributions of the *stx* genes that are carried by cattle STEC strains [117]. For instance, while *stx*1-positive STEC strains tend to colonize cattle more persistently (i.e., for more than 4 months), *stx*2-positive strains only transiently colonize their cattle hosts (i.e., for less than 2 months) [117]. Despite differences in the duration of carriage of STEC strains carrying different *stx* genes, the distribution of specific *stx*-type strains in cattle populations is less clear. Indeed, while several studies have found that there are no significant differences in the prevalence of *stx*2-type STEC strains among cattle populations [117,118,119,120], others have proposed that *stx*2 is 2~4 times more prevalent than its *stx*1-positive counterpart [109,121].

The lack of a clear pattern in the distribution of *stx*1 and *stx*2 across cattle populations could be due to the presence of multiple *stx* genes within a single STEC strain colonizing a cattle host. This can be caused by the influence of multiple *stx*-carrying lysogenic bacteriophages that have introduced multiple *stx* gene variants into a single strain. Reflecting this, in a surveillance study assessing the prevalence of STEC in cattle populations across several provinces in Iran, Jajarmi et al. [118] found that of all STEC strains characterized, 36% and 48% carried only *stx*1 and *stx*2 genes, respectively, while 16% were found to be positive for both *stx*1 and *stx*2 genes. Similarly, Blankenship et al. [121] found that up to 15.9% of STEC strains isolated from cattle populations were exclusively *stx*1-positive, 74.6% were exclusively *stx*2-positive, and 9.5% were positive for both. In some studies, the prevalence of these *stx*1/*stx*2 combination strains appear to be even higher. For instance, Venegas-Vargas et al. [111] found that 29% of cattle STEC strains harbored both *stx*1 and *stx*2 genes, whereas 29% and 42% only possessed one of *stx*1 and *stx2*, respectively. Similarly, Zschock et al. [120] demonstrated that up to 42.7% of cattle STEC were *stx*1/*stx*2 hybrid strains, compared with 11.5% that were *stx*1-exclusive and 45.8% that were *stx*2-exclusive.

Thus, there do not appear to be any specific distribution patterns in the prevalence of *stx*1 and *stx2* across cattle populations. Despite this, however, specific Stx subtypes do appear to be more common in cattle STEC strains than others. Indeed, toxins belonging to the *stx*_1a_, *stx*_1c_, *stx*_2a_, *stx*_2c_, and *stx*_2d_ subtypes are the most prevalent in STEC strains isolated from cattle hosts, with *stx*_1a_ and *stx*_2a_ being the major subtypes. Reflecting this, Capps et al. [122] found that 74.5% of *stx*1-positive cattle STEC strains were specifically *stx*_1a_-positive, compared with 25.5% that produced *stx*_1c_-subtype toxins, whereas 78% of *stx*2-positive cattle STEC produced the *stx*_2a_-subtype, compared with 10.7% and 11.3% that produced *stx*_2c_ and *stx*_2d_-subtype toxins, respectively. Similarly, across 140 *stx*-positive cattle STEC strains, Shridhar et al. [123] found *stx*_1a_ (87.9%) to be the dominant subtype across all *stx*1-positive strains, while out of the *stx*2 subtypes, *stx*_2a_ was predominant (72.5%). Although *stx*_2a_, *stx*_2c_ and *stx*_2d_ appear to be the most prevalent Stx2 toxin subtypes in cattle STEC strains, other studies have been able to identify other Stx2 subtypes in cattle STEC populations, albeit to a lesser degree. For instance, in a collection of cattle STEC strains, Lee et al. [109] found that 53.9% carried toxins belonging to the *stx*_2a_ subtype, whereas 24.5%, 6.9%, 3.9%, and 1.0% carried toxins belonging to the *stx*_2d_, *stx*_2g_, *stx*_2b_, and *stx*_2e_ subtypes, respectively. Regardless of the prevalence of the other subtypes, however, given that the *stx*_2a_ subtype is most commonly associated with severe disease outcomes, its high prevalence across cattle STEC populations supports the notion that cattle represent important animal reservoirs for the emergence of severe STEC infections in humans.

### 4.2. Sheep

Sheep are also an important host reservoir for STEC. Similar to cattle, STEC infection in sheep are commonly asymptomatic [100], though compared with cattle, the prevalence of STEC in sheep populations is higher [124,125,126,127]. For example, in an analysis comparing the prevalence of STEC across different host-reservoir species in Germany, Beutin et al. [124] found that up to 66.6% of sheep carried STEC, whereas only 21.1% of cattle were found to be STEC-positive. Other studies conducted in countries such as Ireland [125], Iran [127], Norway [126], Spain [128], New Zealand [129], Australia [130], the United States [131], and Brazil [132] have also reported high STEC prevalences in sheep populations worldwide, ranging from 45% to as high as 87.6%. Conversely, comparatively fewer studies have reported low STEC prevalences in sheep populations, ranging between 32~36% [120,133,134].

While there does not appear to be an identifiable pattern of distribution of *stx*1- and *stx*2-positive STEC strains within cattle populations, the STEC strains that colonize sheep appear to be more likely to be positive for *stx*1 than *stx*2. For example, Persad et al. [134] found that, among 453 sheep STEC strains, 71% were positive for *stx*1 only, compared with the 11% that were *stx*2-positive only and the 18% that were positive for both. Similarly, Sánchez et al. [128] found that the proportion of *stx*1-postive STEC strains (52.8%) from sheep was markedly greater than that for *stx*2-positive strains (8.4%), though a significant proportion of isolates (38.8%) were found to harbor both *stx*1 and *stx*2 genes.

To date, several Stx subtypes have been identified in sheep, including *stx*_1a_, *stx*_1c_, *stx*_2a_, and *stx*_2b_; of these, it appears that toxins belonging to the *stx*_1c_ and *stx*_2b_ subtypes are the most predominant amongst the STEC strains colonizing sheep populations [125,132,135]. For instance, Wani et al. [136] found that amongst the *stx*1-positive STEC isolates collected from sheep, the *stx*_1c_ subtype was the most prevalent at 73.5%. Furthermore, when considering *stx*2-positive sheep STEC isolates, Martins et al. [132] determined that up to 84.8% *stx*2-positive strains produced toxins belonging to the *stx*_2b_ subtype. Similarly, whole-genomic analyses of 178 STEC strains performed by McCarthy et al. [125] revealed that up to 66% were positive for the *stx*_1c_ subtype, whereas 62% were positive for the *stx*_2b_ subtype. Interestingly, while Han et al. [137] found that up to 61.7% of *stx*1-positive sheep STEC strains belonged to *stx*_1c_, a similar proportion of *stx*2-positive strains carried *stx*_2b_ (52.4%) compared to *stx*_2k_ (47.6%).

Although sheep populations appear to be characterized by high prevalences of STEC carriage, few epidemiological studies have identified sheep products as a major causative agent for human STEC outbreaks [138,139]. This could be due to the finding that the major sheep-associated STEC strains tend to be positive for the milder Stx subtypes, like *stx*_1c_, which are not as common in human patients. Indeed, to date, there have been only a few reported STEC outbreaks caused by contaminated lamb or mutton [138,139].

Importantly, however, while most *stx*1-positive sheep STEC strains appear to be of the *stx*_1c_ subtype and thus may not represent a major public health risk, the high prevalence of *stx*_2b_-positive STEC strains in sheep could still pose a significant risk to human health, especially since *stx*_2b_ is a prominent *stx*2 subtype linked to human infections.

### 4.3. Goat

Another domestic ruminant, goats also serve as important reservoirs of STEC, often exhibiting asymptomatic carriage upon STEC colonization, similar to cattle and sheep. These goat STEC strains can then be transmitted to humans through the consumption of unsterilized goat milk or dairy products [138], leading to human infections. The prevalence of STEC in goat populations varies substantially, with studies reporting STEC prevalences ranging from as low as 9.85% to as high as 80.2%, depending on the farm [127,140,141].

Concerning the distribution of *stx* types in goat STEC strains, several studies have reported an overabundance of *stx*1-positive strains compared with those that carry the *stx*2 gene [142,143]. For instance, Persad et al. [134] found that 79% of STEC strains isolated from goats were *stx*1-positive compared with 18% that were *stx*2 positive and 8% that were positive for both. Similarly, Wiriyaprom et al. [144] found that across several farms in Thailand, the primary *stx* represented was *stx*1, found in 75.44% of STEC strains isolated from goats, compared with 22.81% of goat STEC strains that were *stx*2-positive and 1.75% that carried both *stx*1 and *stx*2. Similarly, Jajarmi et al. [142] found that up to 98.2% of STEC strains colonizing goats are *stx*1 positive, compared with 24.5% that carried *stx*2. Reporting a less drastic difference, Mahanti et al. [145] also reported that goat STEC strains tend to harbour *stx*1 over *stx*2, with prevalences of 72.2% and 58.3%, respectively.

To date, several Stx subtypes have been reported in STEC strains collected from goats, including *stx*_1a_, *stx*_1c_, *stx*_2a_, *stx*_2b_, *stx*_2c_, *stx*_2d_, and *stx*_2k_. Of these, *stx*_1c_ appears to be the predominant subtype carried by goat STEC strains. Indeed, Mahanti et al. [145] found that, of all the Stx subtypes represented across goat STEC strains, 52.8% of strains carried *stx*_1c_, followed by 25% that harbored *stx*_2c_ and 22.2% that carried *stx*_2d_. Furthermore, in a surveillance study conducted by Taghadosi et al. [143], *stx*_1c_ was identified as the most common subtype amongst goat STEC strains, with a prevalence of 63.6%, followed by *stx*_1a_ at 45.5%, *stx*_2c_ at 21.2%, *stx*_2b_ at 18.5%, *stx*_2d_ at 12.1%, and *stx*_2a_ at 3.0%. Similarly, Ndegwa et al. [146] found that amongst goat STEC strains collected over a three-year period in the U.S., *stx*_1c_ was the predominant *stx* subtype, being carried in 47.0% of STEC strains, followed by *stx*_2a_ at 27.5%, *stx*_1a_ at 14.1%, *stx*_2b_ at 13.2%, and *stx*_2d_ at 4.5%.

Although *stx*_1c_ has been consistently found to be the most common Stx subtype in goat STEC strains [140], the dominant subtype within a given goat population can change over time and may also be region-dependent. For instance, Yang et al. [140] found that from 2017 to 2021, *stx*_1c_ was the predominant Stx subtype in STEC strains isolated from several goat farms; however, closer inspection of the yearly trends revealed that by 2021, *stx*_2k_ had become the most common subtype in the goat population in that year. Interestingly, most of the reported *stx*_2k_-positive STEC strains to date appear to have originated from China [140], suggesting that this may be an Stx2 subtype of local concern, especially due to the zoonotic potential of STEC strains carrying this subtype.

### 4.4. Deer

Outside of the common domestic ruminant reservoirs, deer represent important free-living ruminant reservoirs for STEC, especially in countries with large deer populations, such as the U.S. and Canada. The prevalence of STEC appears to vary widely, with studies reporting STEC carriage rates ranging from 9.5% to 93.3% [147,148,149,150,151,152,153]. Interestingly, the wide variance in STEC prevalence does not appear to be species-dependent, as different studies have reported wide differences in STEC prevalence even when studying the same deer species. For instance, Eggert et al. [149] found that 93.3% of red deer in a German national park carried STEC, while Lauzi et al. [151] reported that the STEC prevalence in free-ranging red deer from the Central Italian Alps was only approximately 19.9%. Furthermore, studies examining the carriage of STEC in roe-deer species have ranged from as high as 72.6% [150] and 73.3% [149] to as low as only 16.8% [152]. A similar degree of incongruence has also been observed for STEC prevalence in fallow-deer populations, with estimates ranging from 30.9% to 9.5% [151,153], further demonstrating that the prevalence of STEC in deer can vary extensively.

Regardless of the wide range in STEC prevalence reported across deer populations, stx2 has been consistently found to be the most common *stx* type in deer STEC. Reflecting this, Dias et al. [148] found that all STEC strains collected from a red-deer population were *stx*2-positive. Similarly, Szczerba-Turek et al. [153] found that 94.7% of deer STEC strains carried *stx*2, while only 5.3% were *stx*1-positive. Even studies that have reported a higher proportion of *stx*1, ranging from 21.7% to 38.7%, still found that *stx*2-positive STEC were predominant across all STEC strains isolated from deer, with prevalences ranging from 74.2% to 81.8% [151,152,154].

To date, a variety of Stx subtypes have been identified in deer STEC, including *stx*_1a_, *stx*_1c_, *stx*_2a_, *stx*_2b_, *stx*_2c_, *stx*_2d_, and *stx*_2g_. Of these, *stx*_2b_ appears to be the most commonly observed subtype, either as the lone subtype produced by deer STEC strains or in combination with other subtypes [149,151,155]. For example, Lauzi et al. [151] found that 58.1% of deer STEC strains produced toxins that belonged to the *stx*_2b_ subtype, followed by the *stx*_1c_ and *stx*_2g_ subtypes at 16.1% and 12.9%, respectively. In another study, Szczerba-Turek et al. [153] found that up to 63.1% of deer STEC strains were *stx*_2b_-positive, compared with 15.8% and 10.5% that were *stx*_2a_- and *stx*_2g_-positive, respectively. Importantly, *stx*_2b_ is a common subtype found amongst STEC strains causing human infections, suggesting that STEC strains originating from deer may pose a greater risk to human health than strains derived from other sources. Indeed, several studies have reported contaminated deer meat as the original source of STEC outbreaks in humans [156,157], illustrating the importance of monitoring and evaluating the zoonotic potential of STEC in deer populations.

### 4.5. Swine

While ruminants tend to carry STEC asymptomatically, pigs can experience edema disease upon STEC infection, especially if it occurs shortly after weaning [158]. The STEC associated with edema disease in pigs usually belong to specific serotypes (i.e., O138:H14, O139:H1, and O141:H4) that are not commonly found in humans [159]. Although morbidity can be low, edema disease has a high fatality rate and may cause neurological deficits [160]. The prevalence of STEC in swine has been surveyed by numerous studies. While most studies estimate that roughly 20% to 25% of pigs may carry STEC in a given population [161,162], some have found the prevalence of STEC in pigs to be almost non-existent [163], while others estimate that the prevalence can be as high as 67.7% [164].

Generally, the most common Stx2 subtype in swine STEC has been reported to be *stx*_2e_. Indeed, *stx*_2e_ appears be the most commonly carried Stx2 subtype amongst the STEC strains isolated from pigs, with reported prevalences of 83.0% [165], 85.4% [166], and 98.9% [161], depending on the study. Despite the high degree of consensus shared across these studies, however, other groups have reported other subtypes to be more common [167]. For instance, Arancia et al. [167] reported that in a population of pigs in Italy, 74.2% of STEC strains recovered were positive for *stx*_2a_, compared with only 25.8% for *stx*_2e_.

The consumption of pork has been implicated in several recorded STEC infections in humans [168,169]. For instance, an outbreak in Canada, resulting in 29 patients experiencing gastrointestinal symptoms and 6 developing bloody diarrhea, was found to have been caused by the consumption of contaminated pork products [169]. While pigs may represent a major source of STEC in human populations, the range in disease severity associated with *stx*_2e_-positive STEC strains, from asymptomatic carriage to HUS [170,171], indicates that more research is needed to properly evaluate the human health risks posed by swine-associated STEC strains.

### 4.6. Birds

Various bird species, including chicken, ducks, pigeon, gulls, and geese, have all been reported to carry STEC strains [172,173,174]. Despite this, there are limited studies available on the actual prevalence of STEC amongst bird populations, and it appears that STEC strains are generally rare in birds. Studies that have examined the prevalence of STEC amongst avian hosts, however, have generally focused on pigeons [173,175,176]. Morabito et al. [177], for example, found that 10.8% of pigeon fecal samples collected across three sites in Rome were positive for STEC, with positive carriage rates skewing toward younger birds. Other studies have estimated higher carriage rates amongst pigeon populations. For instance, Grossmann et al. [178] found that up to 66.9% of pigeons carried STEC, whereas Farooq et al. [179] found that 60% of STEC isolated from pigeons carried *stx* genes. Conversely, other studies suggest that pigeons are not important carriers of STEC, as Pedersen et al. [180] were unable to detect any *stx* genes across 406 cloacal swabs collected from pigeons.

Following its original discovery in a pigeon by Schmidt et al. [27] the *stx*_2f_ subtype appears to be the most common Stx subtype in pigeon STEC strains [179,181]. For instance, all of the STEC isolates recovered in the study conducted by Morabito et al. were later found to be *stx*_2f_-positive. Similarly, after isolating STEC from pigeons across four different cities, Badouei et al. [182], found that all the isolates collected produced toxins belonging to the *stx*_2f_ subtype. Beyond *stx*_2f_, however, other studies have reported Stx1 subtypes to be common in pigeon STEC strains. For instance, Grossmann et al. [178] found that while 77.8% (21/27) of pigeon STEC strains carried toxins belonging to the *stx*_2f_ subtype, the remaining 22.2% (6/27) were *stx*1. Moreover, Farooq et al. [179] determined that 55.6% of the STEC strains isolated from domestic pigeons in India carried *stx*1 genes.

At present, no outbreaks caused by STEC originating from pigeons have been reported; however, a case of HUS has been reported in the Netherlands that was caused by an *stx*_2f_-positive O8:H19 *E. coli* strain [183], though it is unclear whether this strain originated from a pigeon host. Despite the lack of known outbreaks associated with pigeon-associated STEC, some studies have commented on the potential of pigeons as a contributor to human STEC outbreaks. For instance, using a whole-genome-based comparative approach, Grande et al. [184] found that STEC strains that cause milder disease in humans could be potentially transmitted from pigeons. Considering that some studies have reported a relatively high proportion of STEC human infections caused by *stx*_2f_-positive strains [185], more research is needed to properly evaluate the role of pigeons as potential reservoirs of *stx*_2f_-positive STEC strains that can be transmitted to and cause disease in human populations.

### 4.7. Other Animals

**Figure 1:**
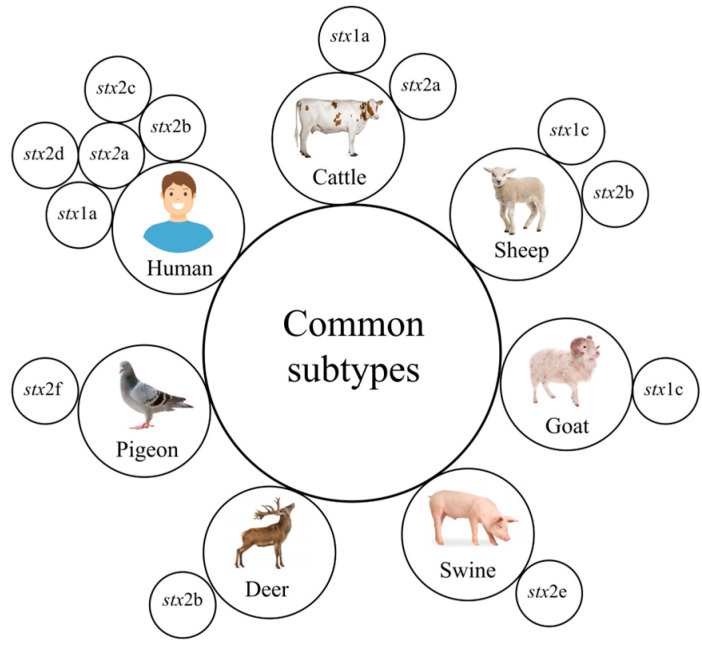
Common Shiga-toxin subtypes in STEC from different host species.

A wide variety of other animal hosts have been reported to carry *stx*-positive *E. coli* strains. For example, Martin et al. [186] recovered an *stx*_2i_-positive STEC strain from Norwegian bivalves. Moreover, Bai et al. [29] originally identified the prototypical *stx*_2h_ subtype from a STEC strain isolated from wild marmots around the the Qinghai–Tibet Plateau in China. Indeed, aside from the major animal reservoirs listed above, a wide range of animal hosts appear to harbor STEC strains, including wild rabbits, horses, donkeys, dogs, cats, coyotes, foxes, and even frogs [124,187,188,189,190]. Figure 1 shows the common Shiga-toxin subtypes in STEC from different host species.

### 4.8. Foods

While food products cannot act as true reservoirs for STEC, they do represent an important medium for STEC transmission via the fecal–oral route. At present, various food products have been reported to be the cause of STEC outbreaks, including beef, lamb, pork, vegetables, juice, and nuts [191]. Specifically, data available from the NCBI pathogen detection database showed that a total of 1923 *E. coli* strains have been isolated from non-host-reservoir sources that carry complete Shiga-toxin sequences (Table 2). Among these STEC strains, beef appears to be the leading isolation source associated, with 1123 strains, while meats from other animals such as swine, sheep, goat, and deer were also found to be prominent isolation sources of STEC (Table 2). In addition to meat, milk and milk products are another important source of STEC, with 93 STEC strains isolated from milk from an unknown host source, 10 from cow milk, 2 from ice cream, and 2 from cream products. Interestingly, the second leading source of STEC appeared to be plant in origin (i.e., produce). As shown in Table 2, 332 STEC strains were recovered from vegetables and 84 were isolated from flour, where the STEC contamination of these plant products may be attributed to the use of animal (i.e., cattle) manure as fertilizer in vegetable and wheat fields. Interestingly, while each food source is generally associated with a myriad of Stx subtypes (Table 2), STEC strains isolated from vegetables appear to be the most diverse with regards to the Stx subtypes represented, likely due to the contamination of vegetables from various sources along each step of the food chain.

## 5. Conclusions

Shiga toxin can be divided into two main groups, Stx1 and Stx2, on the basis of their sequence similarity and key biochemical characteristics. Several bacterial species have been reported to carry *stx* genes; among these, STEC poses the most significant risk to human health due to its widespread prevalence in various animal hosts that have close contact with human populations. Reflecting this, STEC has been found in numerous animal reservoirs including cattle, sheep, goats, pigs, birds, and others, many of which carry STEC asymptomatically. Of these, cattle have been recognized as the main carriers of STEC, representing a significant health risk to humans, as numerous STEC outbreaks originating from contaminated beef and dairy products have been reported each year.

Within Stx1 and Stx2, several subtypes have been identified. Interestingly, the different subtypes appear to vary in their host distribution characteristics, with one or several Stx subtypes being predominantly found in certain host species. Reflecting this, certain Stx subtypes, including *stx*_1a_, *stx*_2a_, *stx*_2b_, *stx*_2c_, and *stx*_2d_, are more commonly associated with human disease than others, while the different subtypes also appear to vary in the severity of diseases that they are associated with. Moreover, given the transmissibility of *stx* genes across different bacterial populations [192] and the growing presence of bacterial strains carrying multiple Stx subtypes simultaneously [193], the virulence potential of bacterial populations capable of producing Shiga toxins may be on the rise. As such, given the wide array of host- and disease-related characteristics associated with Shiga toxin, the rapid and accurate determination of each subtype will be critically important for the proper evaluation of the health risks associated with Stx-producing pathogens.

## Figures and Tables

**Table 1:** Original Shiga toxin subtype information.

*stx* Subtype	NCBI Accession No.	Author (Year)
*stx*_1a_	Sequence available in the article	Calderwood et al. (1987) [4]
*stx*_1c_	AJ312232.1, AJ314838.1, AJ314839.1	Zhang et al. (2002) [19]
*stx*_1d_	AY170851.1	Bürk et al. (2003) [20]
*stx*_1e_	KF926684.1	Probert et al. (2014) [21]
*stx*_2a_	X07865.1	Jackson et al. (1987) [22]
*stx*_2b_	X65949.1	Paton et al. (1992) [23]
*stx*_2c_	M59432.1	Schmitt et al. (1991) [24]
*stx*_2d_	Sequence available in the article	Ito et al. (1990) [25]
*stx*_2e_	Sequence available in the article	Weinstein et al. (1988) [26]
*stx*_2f_	AJ010730.1	Schmidt et al. (2000) [27]
*stx*_2g_	AY286000.1	Leung et al. (2003) [28]
*stx*_2h_	CP022279.1:4122529-4123764	Bai et al. (2018) [29]
*stx*_2i_	FN252457.1	Lacher et al. (2016) [30]
*stx*_2j_	MZ571121.1	Gill et al. (2022) [31]
*stx*_2k_	CP041435.1:4498391-4499631	Yang et al. (2020) [32]
*stx*_2l_	AM904726.1	Koutsoumanis et al. (2020) [33]
*stx*_2m_	WGO76391.1 and WGO76392.1	Bai et al. (2021) [34]
*stx*_2n_	GCA_013342905.2	Lindsey et al. (2023) [35]
*stx*_2o_	MZ229604	Gill et al. (2022) [31]

**Table 2:** Shiga-toxin-gene-positive *E. coli* strains from food sources deposited in NCBI pathogen detection database *.

Food Sources	No. of Strains	Subtypes
Beef	1123	1a, 2c, 2a,1c, 2b, 2e, and 1d
Vegetable	332	1a, 1c, 1d, 2a, 2b, 2c, 2d, 2e, 2f, and 2g
Milk of unknown source	93	1a, 1c, 2a, 2b, 2c, and 2d
Flour	84	1a, 1d, 2a, 2b, 2c, 2e, and 2g
Pork	74	1a, 1c, 2a, 2c, and 2e
Meat of unknown source	68	1a, 1c, 1d, 2a, 2b, 2c, 2d, and 2e
Lamb	38	1a, 1c, 2b, 2c, 2d, and 2e
Cheese	33	1a, 1c, 2a, 2c, and 2e
Deer meat	13	1a, 2b, and 2c
Goat cheese and milk	12	1a, 1c, 2b, and 2c
Hamburger	12	1a and 2c
Milk (cow)	10	1a, 1d, 2a, and 2d
Juice	7	1a
Nuts	6	1a and 2c
Sausage	5	1a and 2c
Chicken	2	2b and 2c
Ice cream	2	2a
Cream (cow)	2	1a
Dessert	2	2c
Seafood	2	2i and 2g
Bear meat	1	1a
Corn product	1	2e
Grain	1	1a

* The database was accessed in August 2023, by which time ~320,000 *E. coli and Shigella* isolates had been deposited into the database.

## Data Availability

All data are available upon request.
